# Serratus Anterior Contraction During Resisted Arm Extension (GravityFit) Assessed by MRI

**DOI:** 10.3389/fphys.2019.01164

**Published:** 2019-09-11

**Authors:** Patrick J. Owen, Timo Rantalainen, Richard A. Scheuring, Daniel L. Belavy

**Affiliations:** ^1^Institute for Physical Activity and Nutrition, School of Exercise and Nutrition Sciences, Deakin University, Geelong, VIC, Australia; ^2^Gerontology Research Center, Faculty of Sport and Health Sciences, University of Jyväskylä, Jyväskylä, Finland; ^3^Johnson Space Center, 2101 NASA Parkway, Houston, TX, United States

**Keywords:** muscle, exercise, rehabilitation, physiotherapy, physical therapy, upper extremity

## Abstract

**Background:**

Scapular stabilization is a common focus of shoulder rehabilitation.

**Objective:**

Examine contraction of serratus anterior during a bilateral arm extension exercise with axial compression using an exercise device (GravityFit) by magnetic resonance imaging (MRI).

**Methods:**

MRI was performed under two conditions: rest and static arm extension with axial compression. Load was set at 20% of age, sex and weight estimated bench press one-repetition maximum. A T2-weighted sequence was used to collect 14 axial images of the upper thoracic spine and shoulder bilaterally. Mean muscle length and thickness were calculated for the whole muscle and in equidistant subregions of the muscle in its anterior (superficial), central and posterior (deep) portions. Adjustment of *p*-values to guard against false positives was performed via the false discovery rate method.

**Results:**

Nine participants without a history of shoulder or spine pathology were included. When compared to rest, arm extension with the exercise device led to 11% increased overall muscle thickness (*P* = 0.038) and 6.1% decreased overall muscle length (*P* = 0.010). Regionally, thickness increased in anterior (superficial, +19%; *P* = 0.040) and central (+17%; *P* = 0.028) portions of the muscle more than posterior (deep, +3.9%, *P* = 0.542).

**Conclusion:**

Contraction of serratus anterior occurred during static arm extension with axial compression produced by a novel exercise approach, as measured via MRI. The activation of serratus anterior differed across its length with greater contraction of the anterior and central portions. This may indicate compartmentalization of function within this muscle. Overall, the proof-of-principle findings justify the use of this exercise approach for the activation of serratus anterior.

## Introduction

The improvement of scapular stabilization is a common focus of shoulder rehabilitation following injury, overuse syndromes and surgery (Duncan and Flowers, 2015). Rehabilitation protocols often place emphasis on targeting the serratus anterior muscle when treating scapula dyskinesia (winged scapula) or following rotator cuff surgery (Duncan and Flowers, 2015). To date, surface electromyography has been primarily used to study serratus anterior to determine which exercises result in optimal activation of this muscle, (Ekstrom et al., 2003, 2005; Ludewig et al., 2004) as well as how the function of the muscle is affected in shoulder pain or injury (Kinsella and Pizzari, 2017). However, surface electromyography is limited in its ability to quantify the posterior portions of serratus anterior located deep to the scapula, which may be more effectively quantified with magnetic resonance imaging (MRI). MRI is a safe methodology for assessing deep and superficial structures of the human body. Moreover, serratus anterior activation has been most commonly demonstrated during complex movements, such as resistance exercises with free weights, (Ekstrom et al., 2003) push-ups (Ludewig et al., 2004) and swimming, (Nuber et al., 1986) with these movements not be feasible across a range of circumstances, including those that involve confined spaces within a hospital rehabilitation setting, such as post-surgical exercise. Therefore, the identification of exercises suitable in these settings are required.

The purpose of the current investigation was to examine whether serratus anterior contraction could be measured by MRI using a novel exercise approach (GravityFit). We hypothesized that static resisted arm extension would lead to serratus anterior contraction, as signified by increased muscle thickness and decreased muscle length compared to rest, in both total and regional portions of the muscle.

## Materials and Methods

This was a cross-sectional study conducted as part of a wider project examining trunk muscle activation using an exercise device, from September 2017 to December 2017 at a medical imaging center in Melbourne, Australia. The study was approved by Deakin University Faculty of Health Human Ethics Advisory Group. All participants gave their informed written consent prior to participation.

Endurance-trained males and females aged 33–55 years were included in the study. Endurance-trained people were examined as our prior experience (Gast et al., 2013; Belavy et al., 2017) with this collective was that their musculature is easier to delineate on MRI and their motor coordination was better than untrained individuals. Individuals were deemed endurance-trained if they participated in at least one half-marathon (approximately. 21 km) distance run in the past year and trained at least twice a week for running for the last 1.5 years or greater. Participants were recruited by online advertisement (Facebook) and direct contact with running clubs in Victoria, Australia. Exclusion criteria included: (1) regular training for other sports more than one day per week within the last year, (2) prior participation in high level sporting codes known to impact spine health [i.e., volleyball, swimming, water polo, weight lifting, rowing, cricket (as a bowler), baseball, gymnastics, American football, equestrian or wrestling], (3) current shoulder, thoracic, neck or lumbar spine pain for which treatment was sought (“treatment” was defined as having seen a physiotherapist, chiropractor, osteopath or medical doctor for the condition), (4) history of shoulder, thoracic, neck or lumbar spine pain for which more than one treatment session was sought, (5) known scoliosis or osteoporosis, and (6) unable to communicate in English. Absolute contraindications to MRI were also implemented (e.g., metal or electrical implants, claustrophobia or possible pregnancy). We estimated that a testing of eight individuals was required to detect an effect size of 0.26, given a power of 0.8, an alpha of 0.05 and correlation of 0.9 between repeated measures (G^∗^Power version 3.1.9.2 was used for these calculations). To account for potential data loss, we included nine individuals in this study.

Subjects were instructed to avoid exercise on the day of testing to control for muscle fatigue. Upon arriving at the medical imaging facility, participants completed questionnaires detailing their demographics. Height and weight were measured using a portable stadiometer and scales.

**FIGURE 1:**
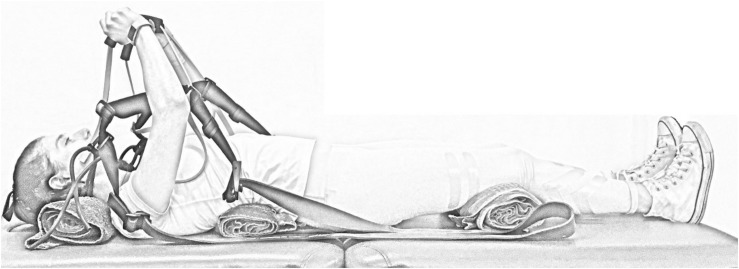
Image of the GravityFit exercise apparatus during compression through the arms during extension.

Magnetic resonance imaging was performed under two conditions: at rest and during static resisted arm extension. A resistance band-based exercise device designed to provide axial compression through the arm during arm extension was used (GravityFit^1^, Peregian Beach, QLD, Australia; Figure 1). At rest, participants were instructed to hold their breath and maintain an isometric arm extension without using resistance bands during scans. During resisted arm extension, participants were asked to hold their breath and maintain an isometric arm extension using resistive bands during scans (Figure 1). The resistance of the bands was an estimated 20% chest press one-repetition maximum based on normative values for age, sex and weight (American College of Sports Medicine, 2017). Resistance was determined by digital force gauge (Digital Scale 40 kg, Rogue, Lawnton, QLD, Australia).

**FIGURE 2:**
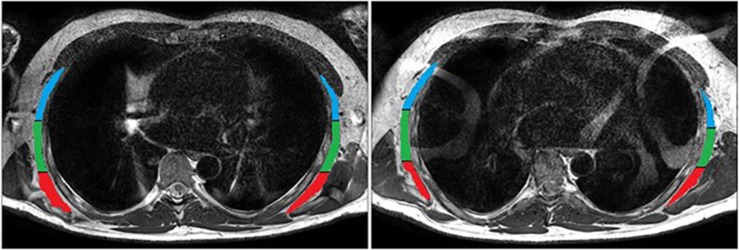
Manual tracing of serratus anterior during rest. Subregions are anterior (superficial; blue), central (green) and posterior (deep; red).

**FIGURE 3:**
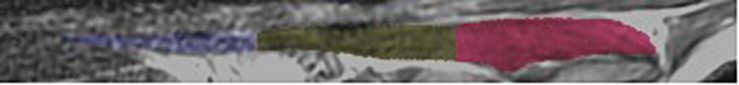
Serratus anterior during rest (top) and contraction (bottom) after the removal of muscle curvature and division into three equidistant sub-regions: anterior (superficial; blue), central (green) and posterior (deep; red).

To quantify muscle morphology on a 3T Phillips Ingenia scanner (Amsterdam, Netherlands) a T2-weighted sequence (thickness, 3 mm; interslice distance, 5 mm; repetition time, 2840 ms; echo time, 60 ms; field of view, 347 × 347 mm, 768 × 768 pixels) was used with spinal coils to collect 14 axial images encompassing the volume of the serratus anterior (Figure 2). Data were exported for offline processing. To ensure blinding of the examiner, each subject was assigned a random numeric code^2^. ImageJ 1.48v^3^ was used to perform all quantitative MRI measures. After tracing around the serratus anterior (Figure 2), a custom written ImageJ plugin (ROI Analyzer^4, 5^) was used to fit a fourth order polynomial to the region of interest and the curvature from the muscle was removed (Figure 3). Maximum and mean serratus anterior length and thickness were calculated for the overall muscle. The muscle was then separated into three equidistant subregions based on maximum muscle length and mean muscle length and thickness were calculated for each subregion. The muscle was measured from the inferior angle of the scapula superiorly up until the point where separation of this muscle from subscapularis was no longer evident. Data were then averaged across slices, as well as between the left and right sides. The reliability of the outcome measure is excellent, with an ICC_2,1_ of 0.95 for serratus anterior muscle thickness [standard error of the measurement (SEM): 0.4 mm] and 0.93 for serratus anterior muscle length (SEM: 1.8 mm).

All statistical analyses were conducted using Stata statistical software version 15 (College Station, TX, United States). Linear mixed models with random effects were used to evaluate differences between rest and contraction conditions. To mitigate the risk of type I errors, all *P*-values were adjusted by the false discovery rate method (Benjamini and Hochberg, 1995). An alpha-level of 0.05 was adopted for all statistical tests.

## Results

Nine participants (7 males and 2 females) were measured. On average, participants age, height, weight and body mass index were 46 years (range, 36–56 years), 172.5 cm (range, 160.9–185.5 cm), 73.5 kg (range, 61.0–90.2 kg) and 24.6 kg/m^2^ (range, 22.1–27.2 kg/m^2^), respectively.

**FIGURE 4:**
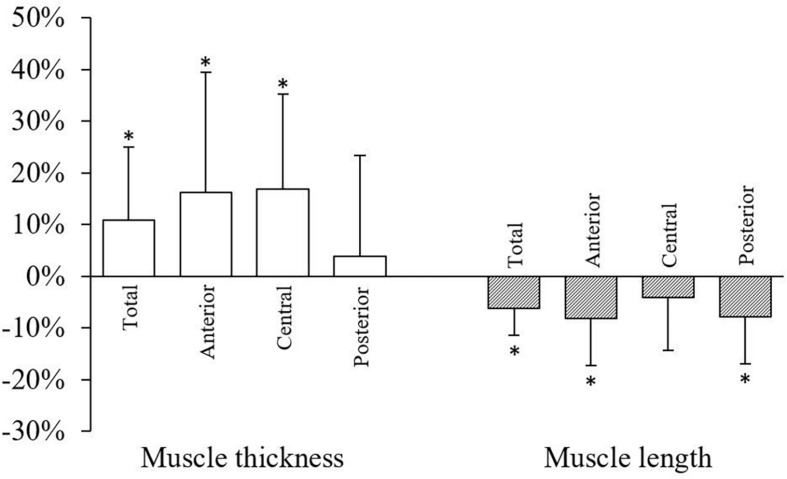
Changes in serratus anterior total muscle thickness (mean) and length (maximum) and subregion muscle thickness (mean) and length (mean) during contraction compared to rest. Data are mean percent change (standard deviation). ^∗^*P* < 0.05.

Serratus anterior length and thickness data during both conditions are presented in Table 1. When compared to rest, overall muscle thickness (mean) increased and length (maximum) decreased by 11% (*t* = 2.35, *P* = 0.019, apparent power = 69%) and 6.1% (*t* = −3.44, *P* = 0.001, apparent power = 80%), respectively (Figure 4). During contraction, mean muscle thickness increased compared to rest in the anterior (19%; *t* = 2.25, *P* = 0.024, apparent power = 99%) and central (17%; *t* = 2.63, *P* = 0.009, apparent power = 90%) subregions, but not posterior (Figure 4). Moreover, mean muscle length decreased during contraction in the anterior (8.2%; *t* = −2.76, *P* = 0.006, apparent power = 99%) and posterior (7.7%; *t* = −2.54, *P* = 0.011, apparent power = 85%) subregions compared to rest. Similar findings were not observed in the central subregion (Figure 4). The application of the false discovery rate did not alter the significance of any outcomes.

**TABLE 1:** Serratus anterior muscle thickness and length during rest and contraction.

**Parameter**		**Rest, mm**	**Contract, mm**	**Change, mm**	**Cohen’s *d***
Muscle thickness	Mean	6.4 (1.4)	7.1 (1.8)	0.7(0.9)^∗^	0.464
	Max	11.0 (2.2)	11.8 (2.8)	0.9 (1.3)	0.342
Anterior muscle thickness	Mean	4.3 (0.9)	5.1 (1.2)	0.7(1.0)^∗^	0.697
Central muscle thickness	Mean	7.1 (1.7)	8.3 (2.2)	1.2(1.3)^†^	0.586
Posterior muscle thickness	Mean	7.7 (2.0)	8.0 (2.5)	0.3 (1.5)	0.133
Muscle length	Mean	93.5 (12.1)	90.6 (11.7)	−2.9(10.7)	–0.244
	Max	147.2 (17.8)	138.2 (16.5)	−9.0(7.8)^∗^	–0.522
Anterior muscle length	Mean	34.1 (4.8)	31.3 (3.1)	−2.8(3.1)^†^	–0.690
Central muscle length	Mean	39.8 (5.5)	38.2 (4.8)	−1.6(4.1)	–0.318
Posterior muscle length	Mean	36.2 (4.8)	33.4 (5.2)	−2.8(3.3)^∗^	–0.554

*Data are mean (standard deviation). ^∗^*P* < 0.05, ^†^*P* < 0.01.*

## Discussion

We showed here that during an arm extension exercise with axial compression, shortening and thickening of the serratus anterior occurs muscle bilaterally, as measured via MRI. Increased muscle thickness was greatest in the anterior (superficial) and central portions than the posterior (deep) portion of the muscle.

The serratus anterior muscle is often a focal point during shoulder rehabilitation, however the optimal exercise approach to target this muscle during MRI remains unknown. The application of axial loading through the arms in our study, provided by the exercise device examined, led to serratus anterior contraction during static arm extension. Traditionally, activation of serratus anterior has relied upon approaches that may not be suitable for MRI, including manual muscle testing, (Ekstrom et al., 2005) free weights (Ekstrom et al., 2003) and swimming, (Nuber et al., 1986). Some of these exercise approaches may also pose challenges in the clinical setting due to confirmed spaces, such as within a hospital bed post-surgery. The exercise approach examined in our study, mainly due to the compact design and ease of use of the device, may allow for the activation of serratus anterior in these settings. Moreover, the exercise device in our study allowed for the modification and quantification of resistive load between and within participants, respectively. The findings provide proof-of-principle justification for the use of this exercise device for the activation of serratus anterior during MRI.

Our data may also provide some insight into differential activation of serratus anterior. Differential activation of a muscle has been referred to as compartmentalization of muscle function, (English et al., 1993) specifically that sub-units of a given muscle have different functional roles. This has been observed in other muscles. For example, the adductor magnus muscle in the thigh consists of both an “extensor portion” and an “adductor portion” with the former innervated by the sciatic nerve and the latter by the obturator nerve (Bardeen, 1906). In lower vertebrates, this “extensor portion” is actually a separate muscle grouped with the hamstring muscles, (Howell, 1938) whereas in human embryos, these two sections of the adductor magnus muscle develop separately and later fuse (Bardeen, 1906). The transversus abdominis muscle of the trunk shows similar anatomical (Urquhart et al., 2005a) and functional (Urquhart and Hodges, 2005; Urquhart et al., 2005b) compartmentalization. In this work we saw greatest increases in muscle thickness in the central portion of serratus anterior. Muscle thickness has been shown to be associated with force production (Muraki et al., 2013; Akagi et al., 2018). Another study using electromyography also showed evidence of compartmentalization of serratus anterior (Ekstrom et al., 2004). Whilst preliminary, our data provide additional evidence for compartmentalization of serratus anterior function which could be investigated in future work for determining optimal activation and rehabilitation exercise strategies for this muscle.

To our knowledge, the only other published method that has examined serratus anterior contraction using MRI utilized a functional MRI technique that quantified intra-muscular water content following a 3-rep × 1-minute hold time (30-second rest between reps) isometric shoulder abduction exercise protocol at 20% maximum voluntary contraction (Sheard et al., 2012). The study examined changes in muscle T2 values, which is a measure of free-water in a tissue. The study demonstrated activation of serratus anterior in this exercise in a cohort of healthy participants examined (Sheard et al., 2012). Notably, the exercise protocol within this previous study likely relied heavily upon the anaerobic glycolytic energy system, which can require 30–60 min recovery following fatigue; (American College of Sports Medicine, 2017) thus the feasibility of this protocol for examining the effects of multiple exercises in succession, or for investigating intra-muscular differences in activation, may be limited. Our method, however, utilized brief approximately 30-second bouts of exercise, which could allow for a battery of exercises to be examined and implemented over a shorter timeframe. Thus, the approach we present may be more feasible for examining optimal activation of serratus anterior and also functional compartmentalization within the muscle.

It is appropriate to discuss some of the limitations of the current study, which primarily stem from the quantification of serratus anterior via a novel MRI technique. The acquisition of images and subsequent analyses incur high financial costs and require appropriately trained individuals. In terms of the exercise approach, a wider range of maneuvers and loading levels, to determine optimal activation of the muscle, should be considered. Notably, muscle contraction was inferred from changes in muscle length and thickness, which may limit the validity of our observations. We did not use MRI T2-weighted signal intensity or T2 for assessing muscle contraction as in low intensity contractions, there is insufficient change in blood flow to result in detectable changes in muscle T2. Also, measurement of muscle T2 requires a long (>5 min) scanning sequence and this greatly exceeds feasible breath-hold time. The use of T2-weighted signal intensity for estimating changes in hydration is inappropriate as T2-weighted signal intensity is influenced by a number of factors that are unrelated to the participant (e.g., scaling and re-scaling factors from the MR software). For this reason, we focused on changes in muscle morphology when assessing muscle contraction. It is also important to note that we only considered healthy participants and thus whether similar observations of serratus anterior can be obtained in those who have specific shoulder pathologies is unknown. Finally, as only one study to date (McKenna et al., 2018) has examining serratus anterior muscle in people with and without shoulder pathology, and found no significant difference between the groups, we cannot yet speculate on clinical significance. Future work could extend this to populations with shoulder pathology.

## Conclusion

In conclusion, this study demonstrated the ability of a novel exercise approach to contract serratus anterior. Notably, the exercise examined led to whole muscle and regional (i.e., anterior and central) contractions of serratus anterior. These proof-of-principle findings justify the use of this exercise device for the activation of serratus anterior, for example in shoulder rehabilitation protocols.

## Data Availability

The datasets for this manuscript are not publicly available because of participant privacy. Requests to access the datasets should be directed to DB, d.belavy@deakin.edu.au.

## Ethics Statement

This study was carried out in accordance with the recommendations of Australian Code for Responsible Conduct of Research (2007) and National Statement on Ethical Conduct of Human Research (2007). The protocol was approved by the Deakin University Human Research Ethics Committee.

## Author Contributions

All authors designed the study, interpreted the data, revised the manuscript, and approved the final version of the manuscript. PO and DB conducted the study and collected the data. PO, TR, and DB analyzed the data. PO drafted the manuscript.

## Conflict of Interest Statement

The project funder, an industrial entity with commercial interest in the exercise device, was involved in project design and approved the final version of the submitted manuscript.

## Footnotes

1 www.gravityfit.com

2 www.random.org

3 http://rsb.info.nih.gov/ij/

4 https://github.com/tjrantal/RoiAnalyzer

5 https://sites.google.com/site/daniellbelavy/home/roianalyser
